# Supplementary material for: Exploring the Genetic and Clinical Landscape of Dedifferentiated Endometrioid Carcinoma
Source: Int J Mol Sci. 2025 Apr 27;26(9):4137. doi: 10.3390/ijms26094137 (PMC12071752; doi:10.3390/ijms26094137)
Supplement: Supplementary file 1 [file ijms-26-04137-s001.zip › Supplementary Figure.pdf]

Supplementary Figure S1. Kaplan–Meier analysis of progression-free survival and overall survival

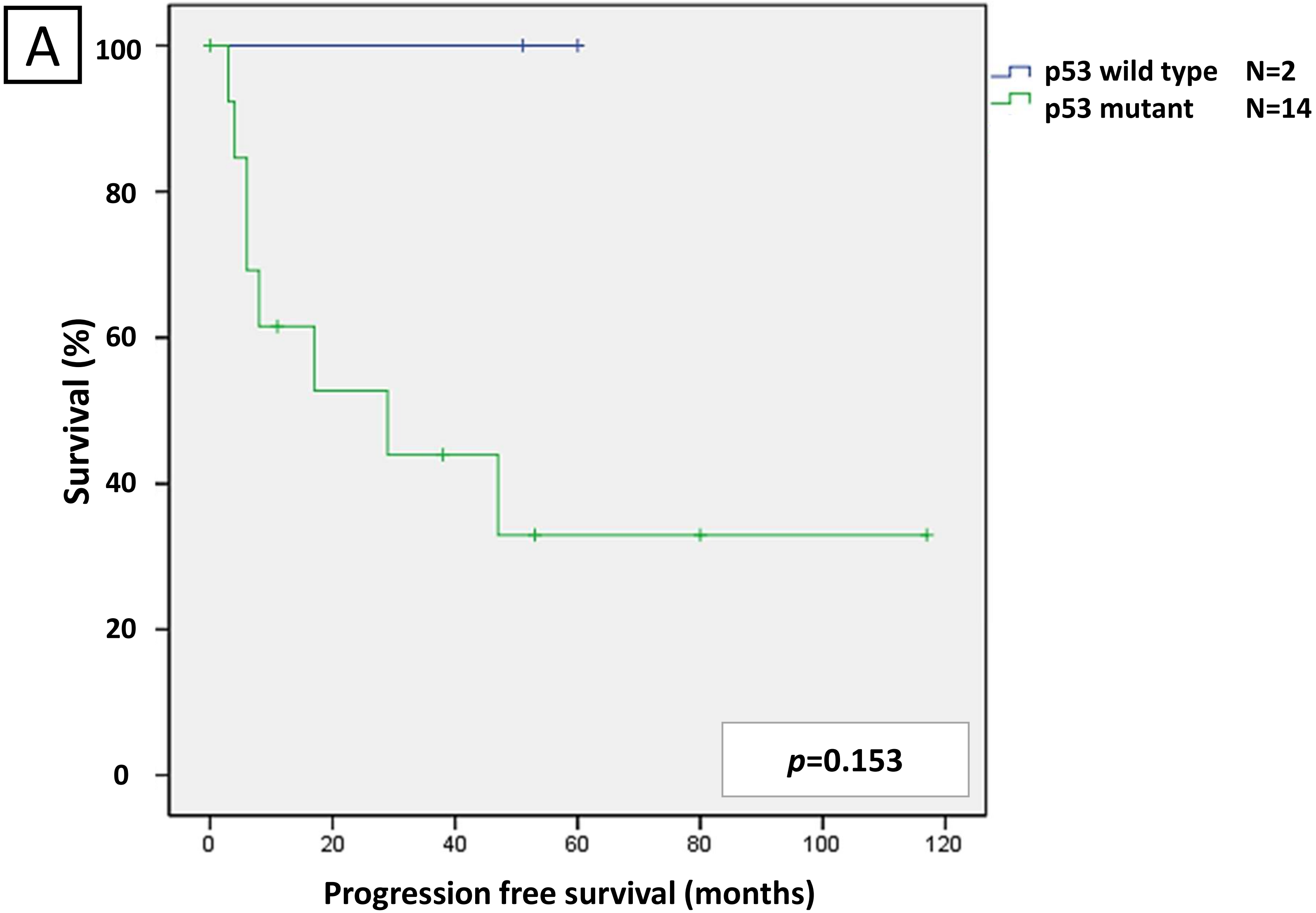

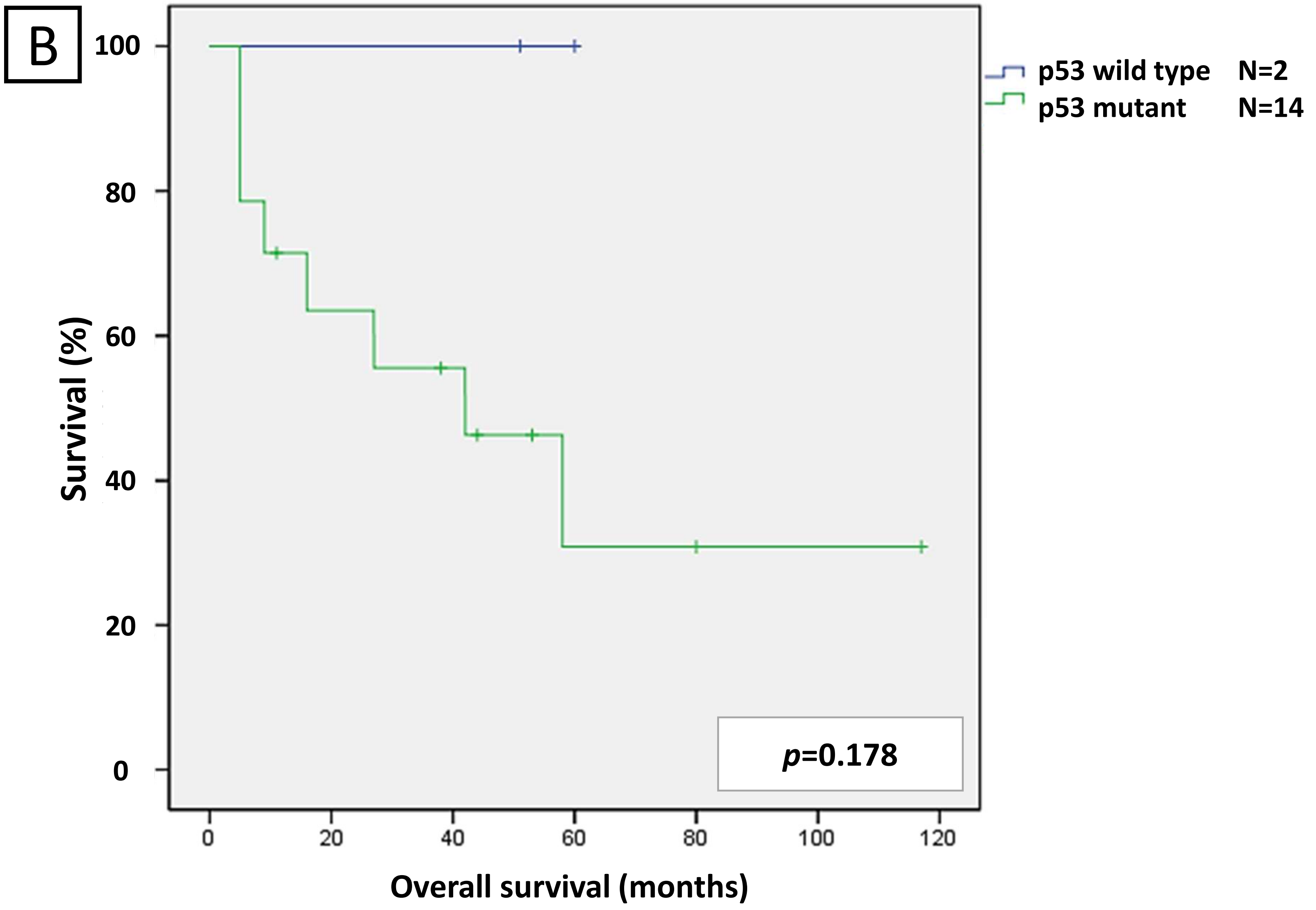
B
100
80
60
40
20
0
Survival (%)
0
20
40
60
80
100
120
Overall survival (months)
p53 wild type N=2
p53 mutant N=14
p=0.178

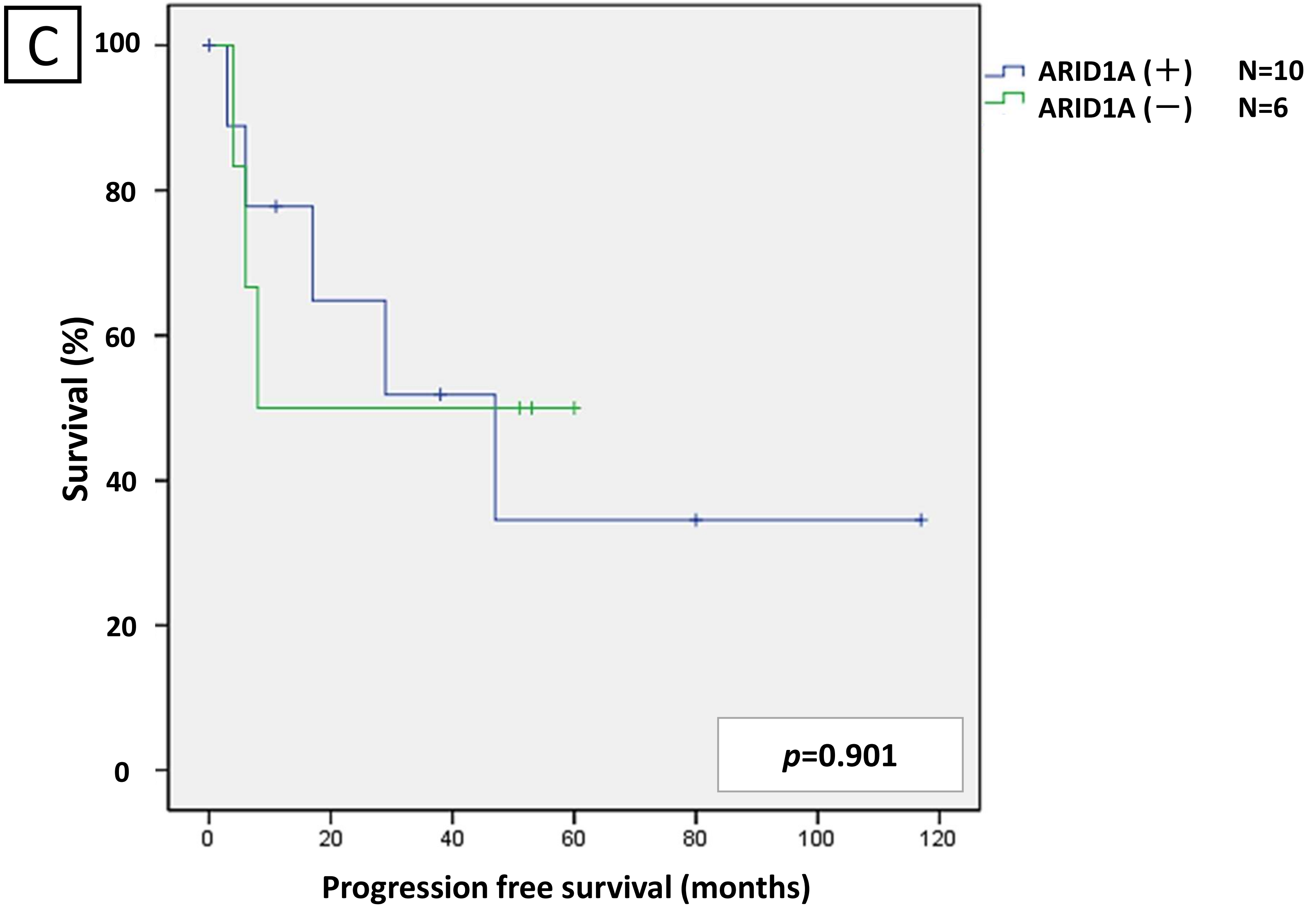
C
100
80
60
40
20
0
Survival (%)
0
20
40
60
80
100
120
Progression free survival (months)
ARID1A (+) N=10
ARID1A (−) N=6
p=0.901

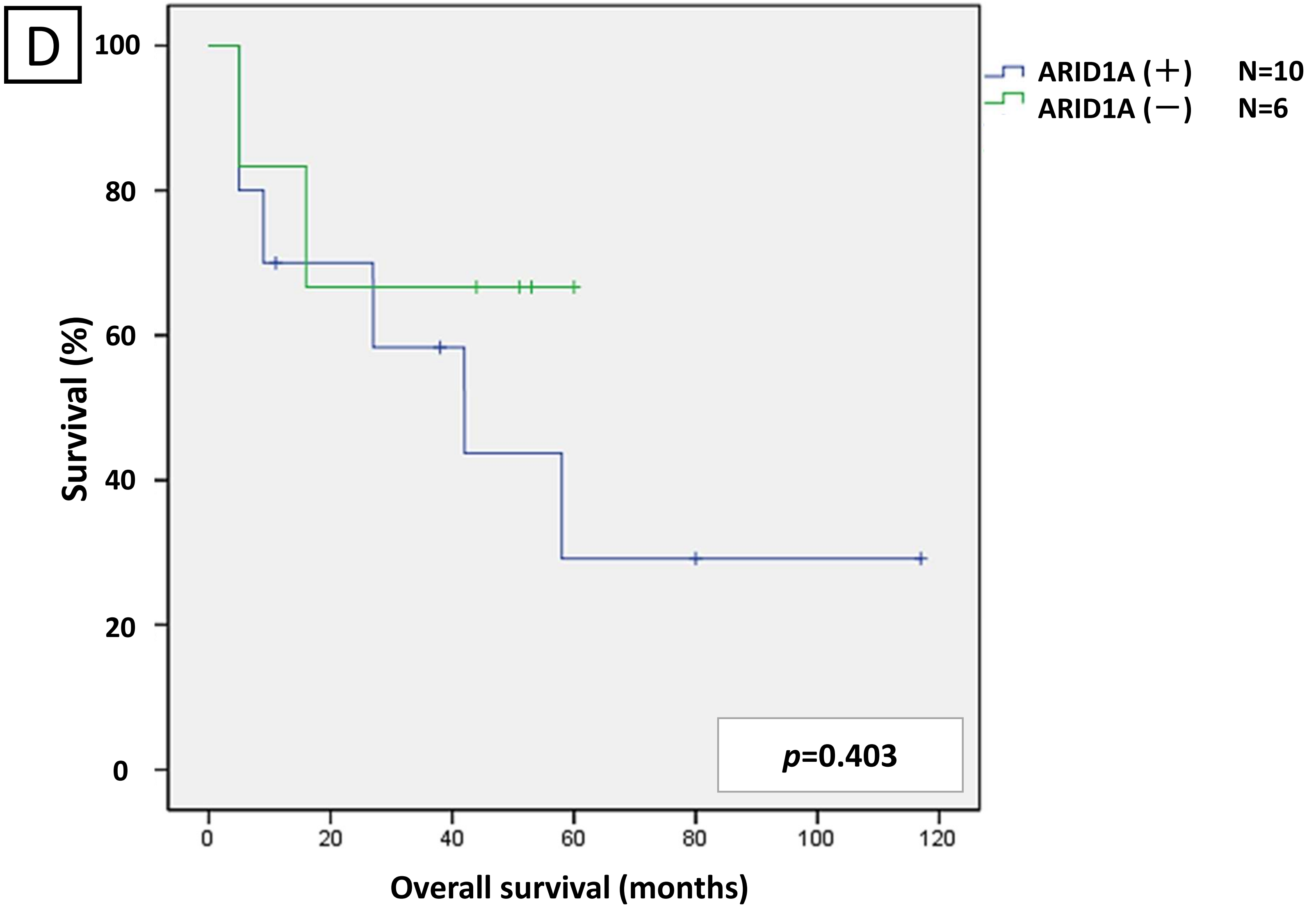
D
Survival (%)
100
80
60
40
20
0
0
20
40
60
80
100
120
Overall survival (months)
ARID1A (＋) N=10
ARID1A (－) N=6
p=0.403

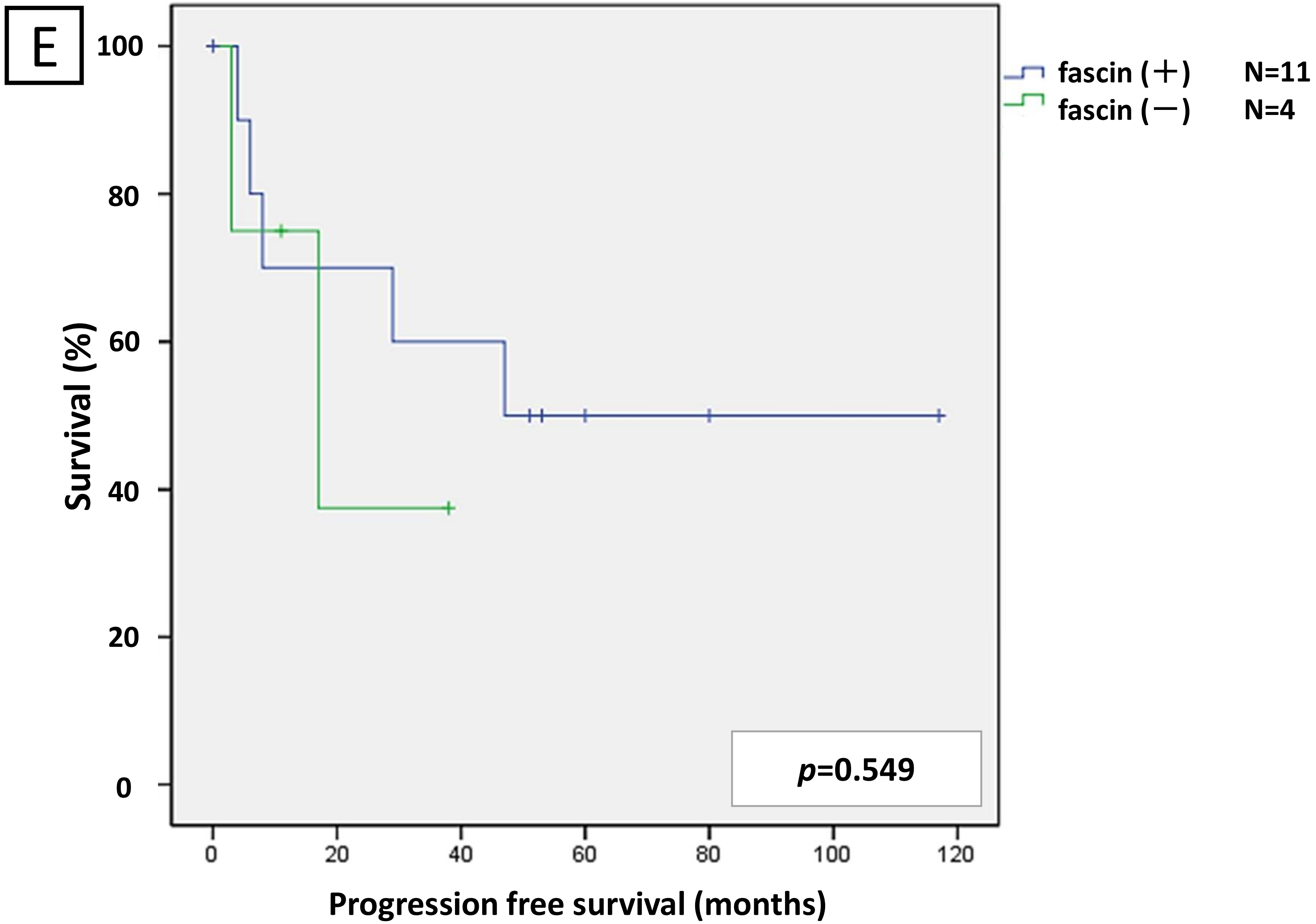
E
100
80
60
40
20
0
Survival (%)
fascin (＋) N=11
fascin (－) N=4
p=0.549
0
20
40
60
80
100
120
Progression free survival (months)

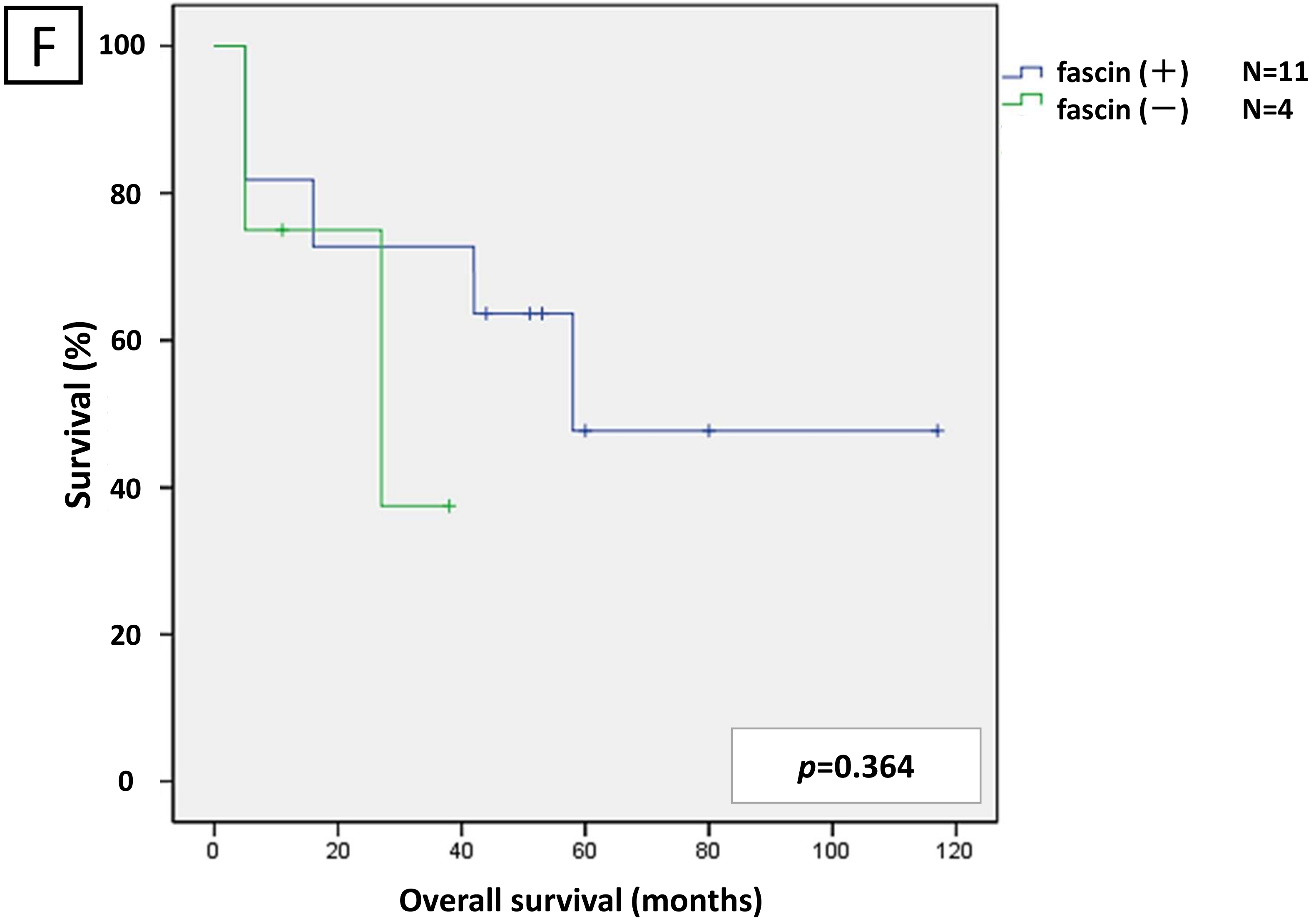
F
100
80
60
40
20
0
Survival (%)
fascin (+) N=11
fascin (−) N=4
p=0.364
0
20
40
60
80
100
120
Overall survival (months)

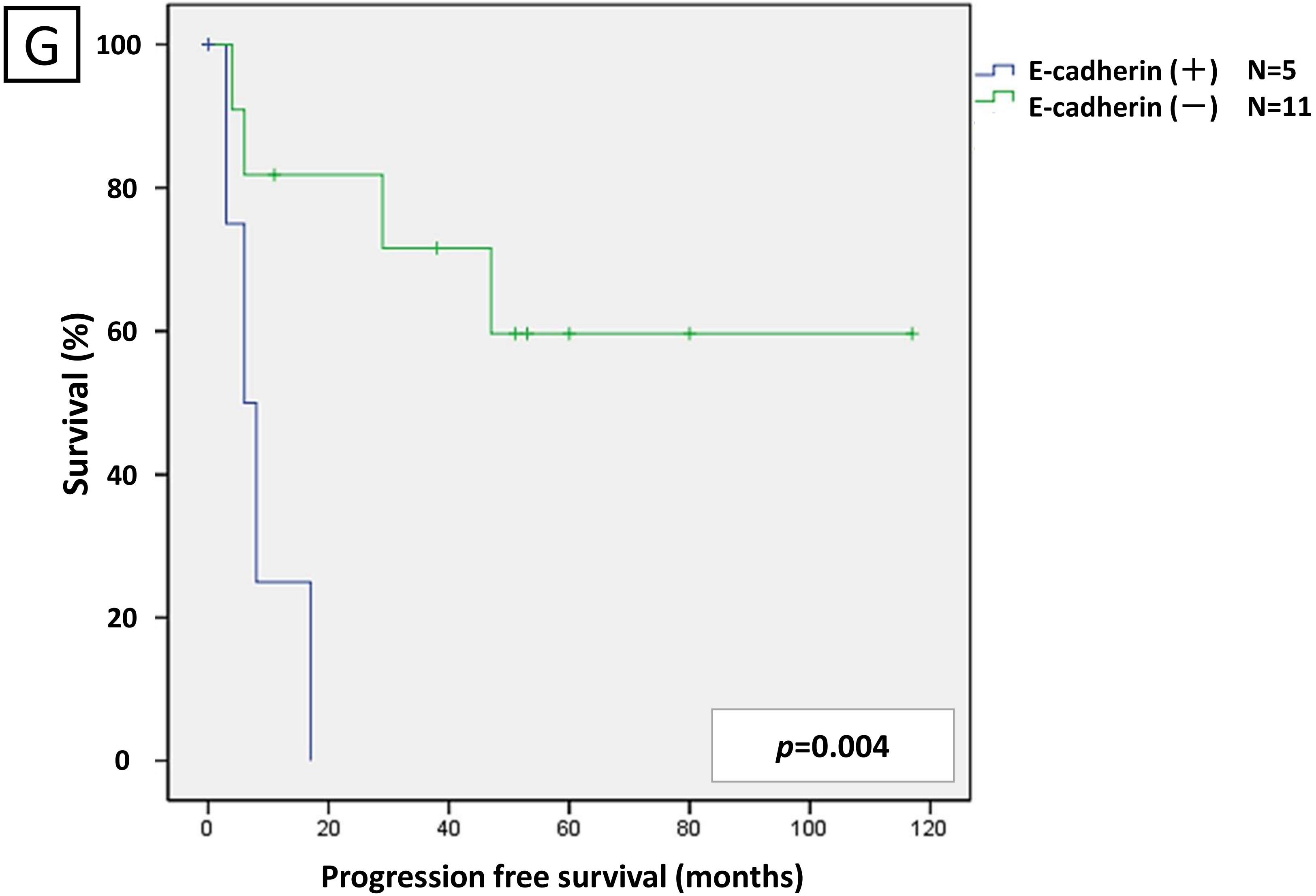

G
100
80
60
40
20
0
Survival (%)
0
20
40
60
80
100
120
Progression free survival (months)
E-cadherin (＋) N=5
E-cadherin (－) N=11
p=0.004

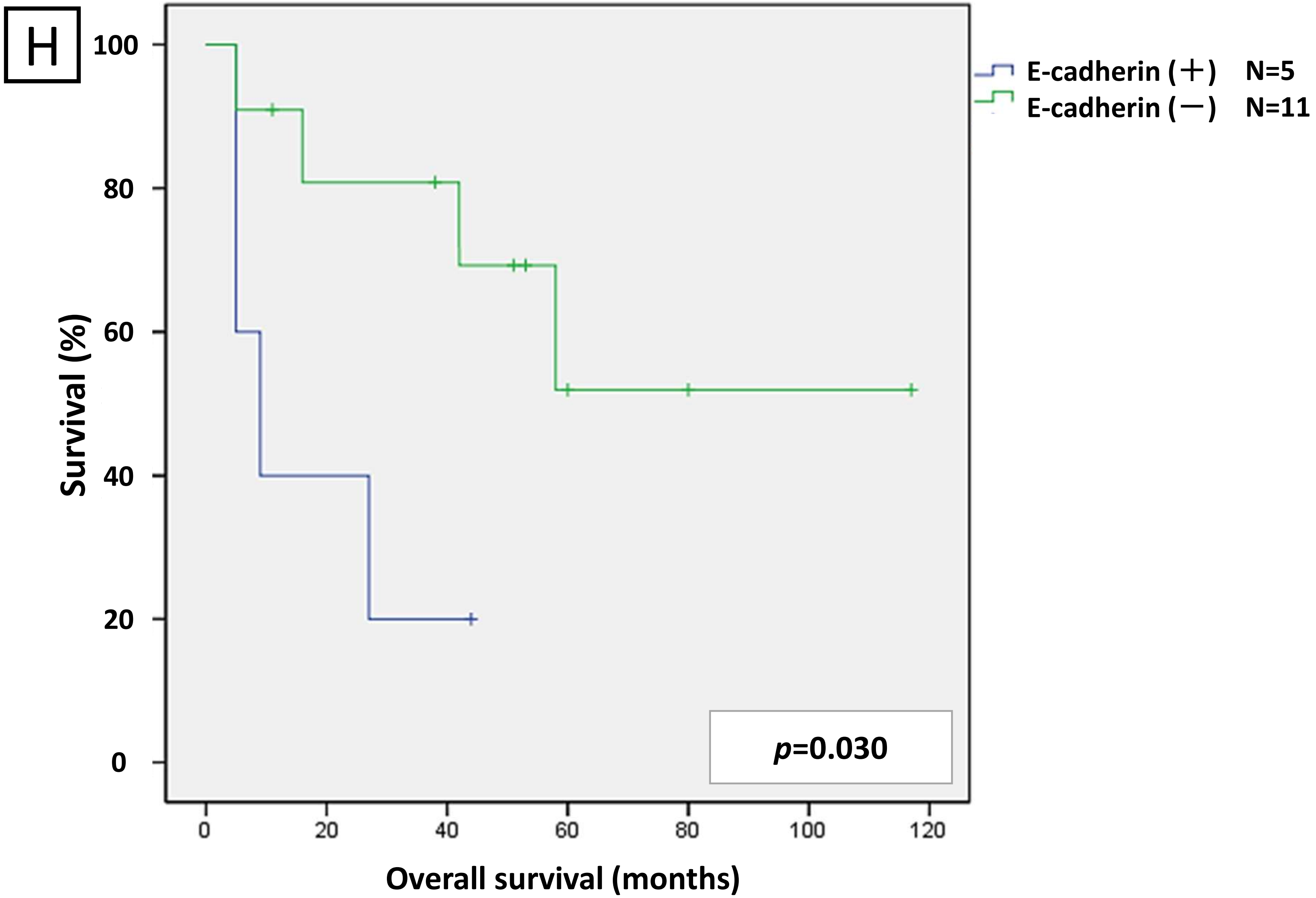

H
100
80
60
40
20
0
Survival (%)
E-cadherin (＋) N=5
E-cadherin (－) N=11
p=0.030
0
20
40
60
80
100
120
Overall survival (months)

Supplementary Figure S2. Mutation signatures

A

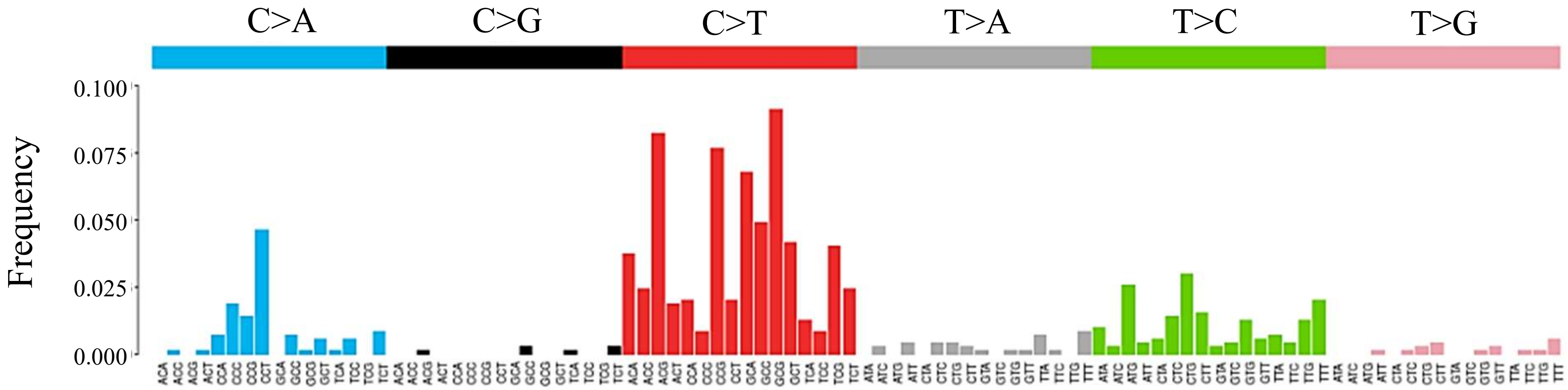

Cosine similarities with COSMIC mutation signatures

Rank	Signature	Similarity	Cancer type
1	Signature 6	0.961	Signature 6 has been found in 17 cancer types and is most common in colorectal and uterine cancers. In most other cancer types, Signature 6 is found in less than 3% of examined samples.
2	Signature 1	0.853	Signature 1 has been found in all cancer types and in most cancer samples.
3	Signature 14	0.774	Signature 14 has been observed in four uterine cancers and a single adult low-grade glioma sample.
4	Signature 20	0.759	Signature 20 has been found in stomach and breast cancers.
5	Signature 15	0.733	Signature 15 has been found in several stomach cancers and a single small cell lung carcinoma.

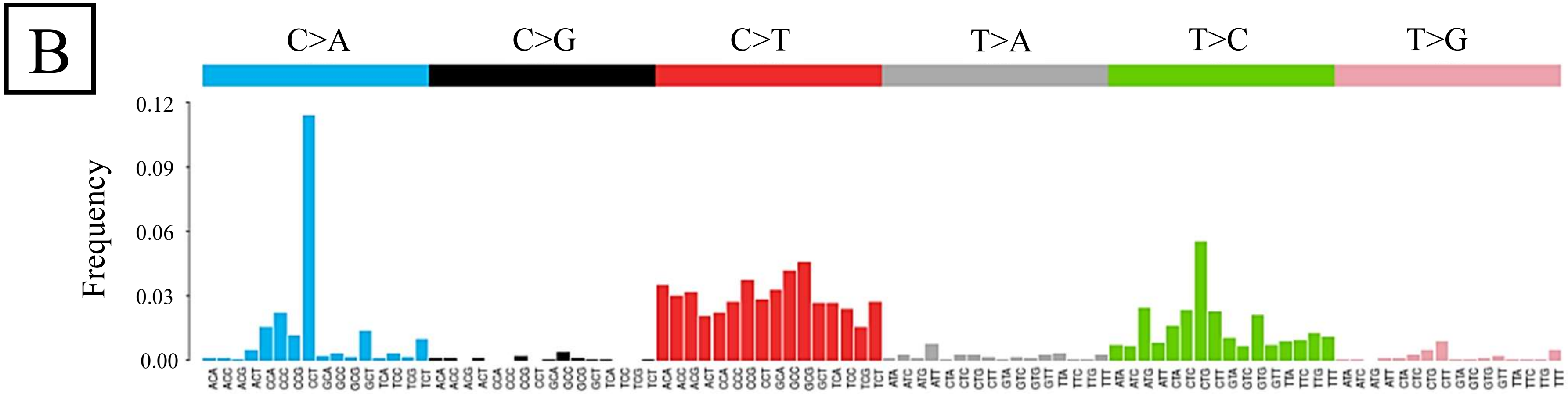

Cosine similarities with COSMIC mutation signatures

Rank	Signature	Similarity	Cancer type
1	Signature 20	0.909	Signature 20 has been found in stomach and breast cancers.
2	Signature 14	0.821	Signature 14 has been observed in four uterine cancers and a single adult low-grade glioma sample.
3	Signature 6	0.747	Signature 6 has been found in 17 cancer types and is most common in colorectal and uterine cancers. In most other cancer types, Signature 6 is found in less than 3% of examined samples.
4	Signature 5	0.658	Signature 5 has been found in all cancer types and most cancer samples.
5	Signature 19	0.605	Signature 19 has been found only in pilocytic astrocytoma.

C

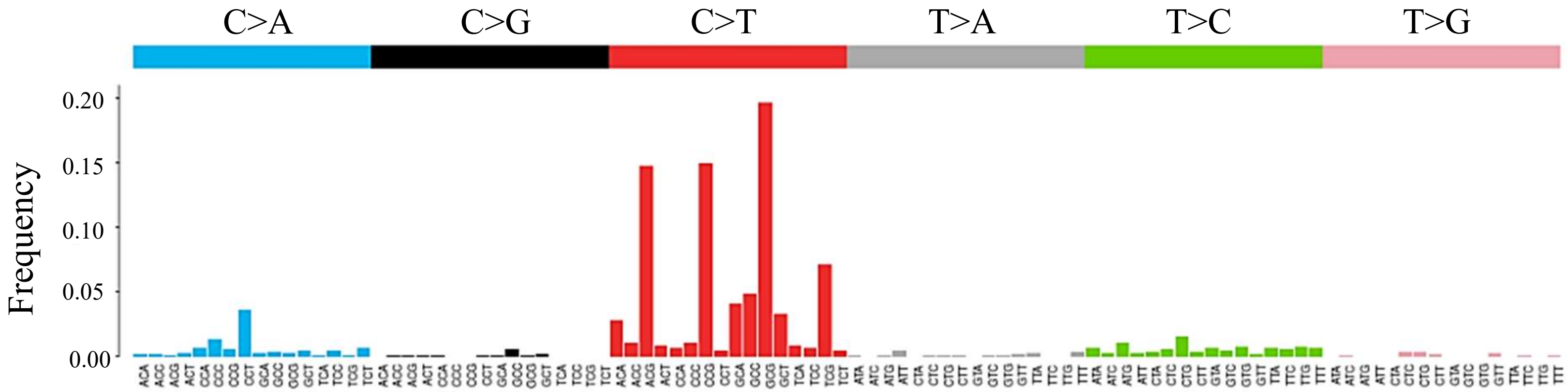

Cosine similarities with COSMIC mutation signatures

Rank	Signature	Similarity	Cancer type
1	Signature 6	0.936	Signature 6 has been found in 17 cancer types and is most common in colorectal and uterine cancers. In most other cancer types, Signature 6 is found in less than 3% of examined samples.
2	Signature 1	0.924	Signature 1 has been found in all cancer types and in most cancer samples.
3	Signature 15	0.711	Signature 15 has been found in several stomach cancers and a single small cell lung carcinoma.
4	Signature 14	0.649	Signature 14 has been observed in four uterine cancers and a single adult low-grade glioma sample.
5	Signature 20	0.482	Signature 20 has been found in stomach and breast cancers.

D

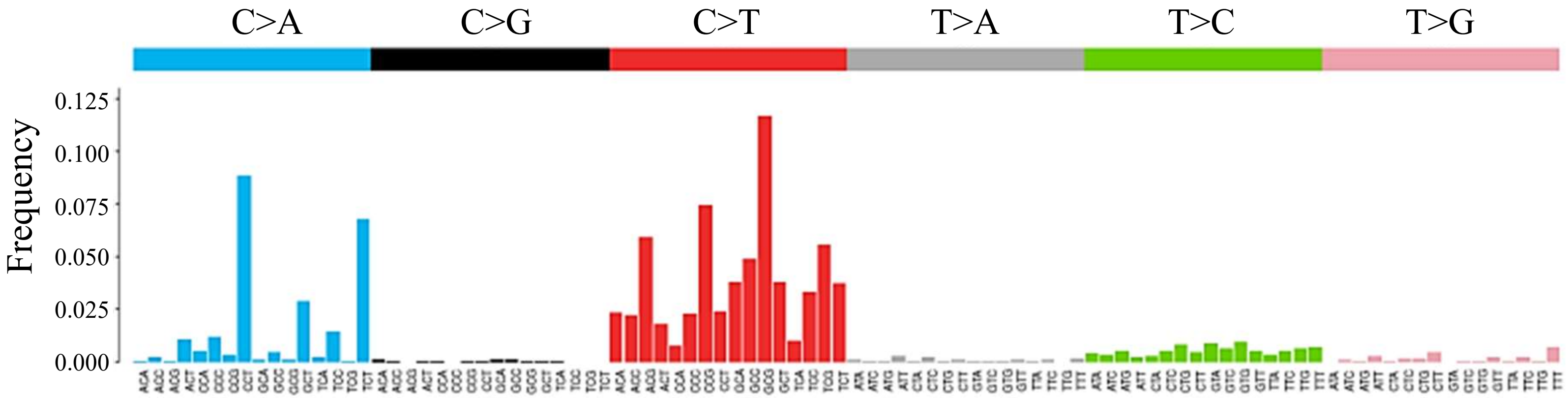

Cosine similarities with COSMIC mutation signatures

Rank	Signature	Similarity	Cancer type
1	Signature 14	0.902	Signature 14 has been observed in four uterine cancers and a single adult low-grade glioma sample.
2	Signature 6	0.888	Signature 6 has been found in 17 cancer types and is most common in colorectal and uterine cancers. In most other cancer types, Signature 6 is found in less than 3% of examined samples.
3	Signature 1	0.767	Signature 1 has been found in all cancer types and in most cancer samples.
4	Signature 15	0.744	Signature 15 has been found in several stomach cancers and a single small cell lung carcinoma.
5	Signature 20	0.667	Signature 20 has been found in stomach and breast cancers.

Supplementary Figure S3. Copy number alteration plot

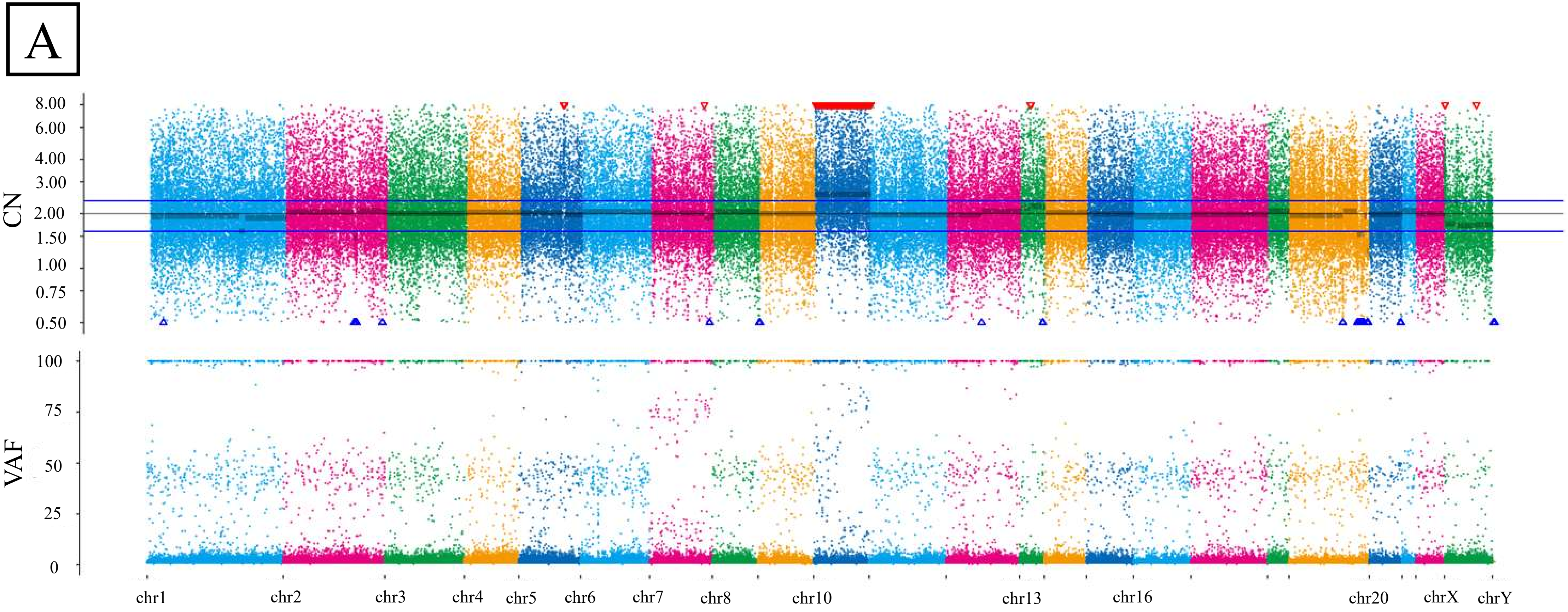

B

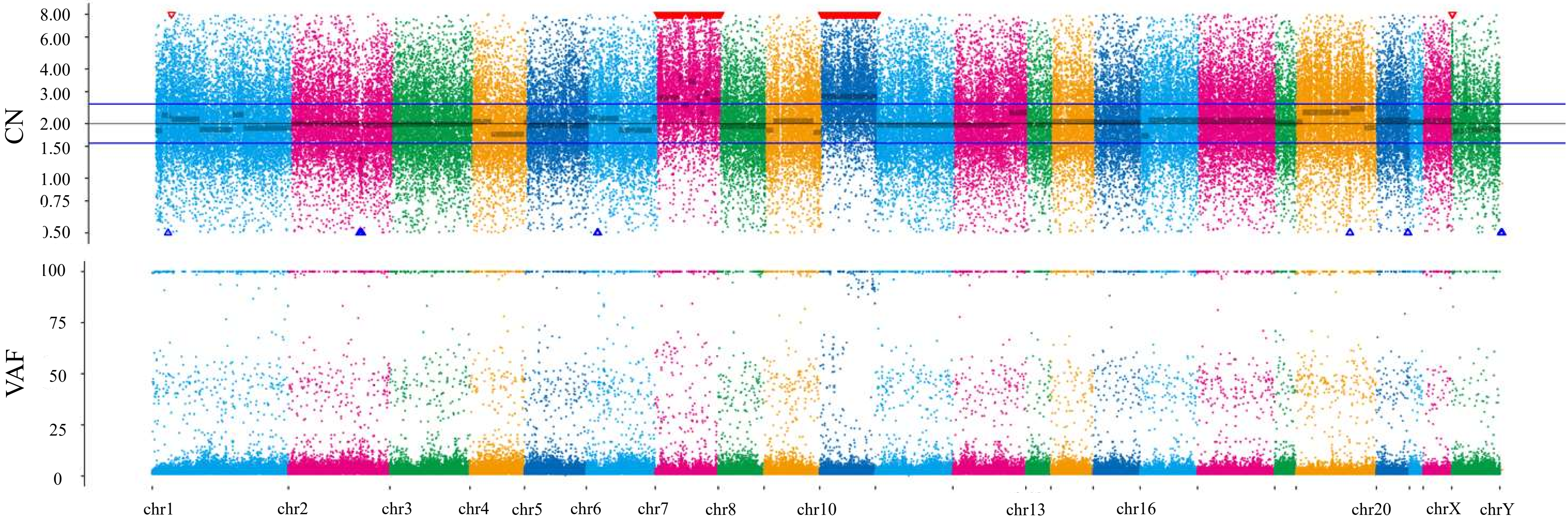

CN
8.00
6.00
4.00
3.00
2.00
1.50
1.00
0.75
0.50
VAF
100
75
50
25
0
chr1
chr2
chr3
chr4
chr5
chr6
chr7
chr8
chr10
chr13
chr16
chr20
chrX
chrY

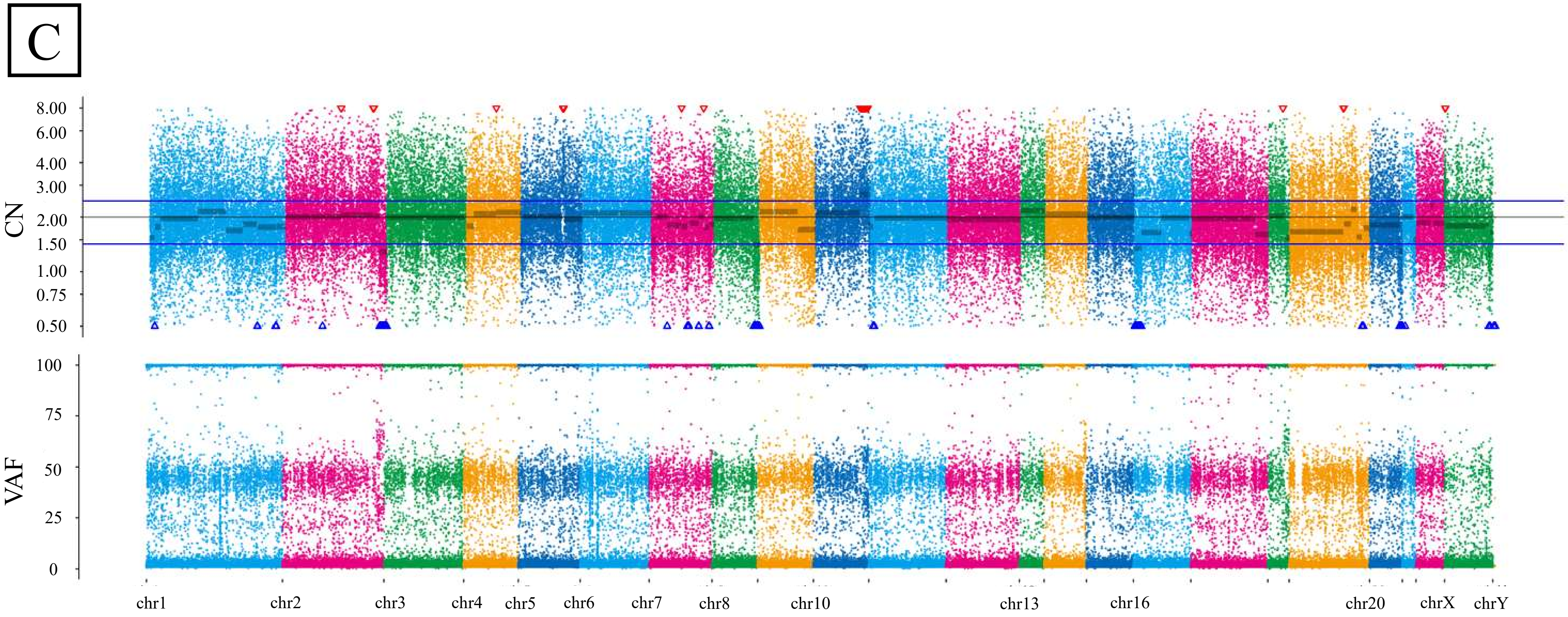
C
CN
8.00
6.00
4.00
3.00
2.00
1.50
1.00
0.75
0.50
VAF
100
75
50
25
0
chr1
chr2
chr3
chr4
chr5
chr6
chr7
chr8
chr10
chr13
chr16
chr20
chrX
chrY

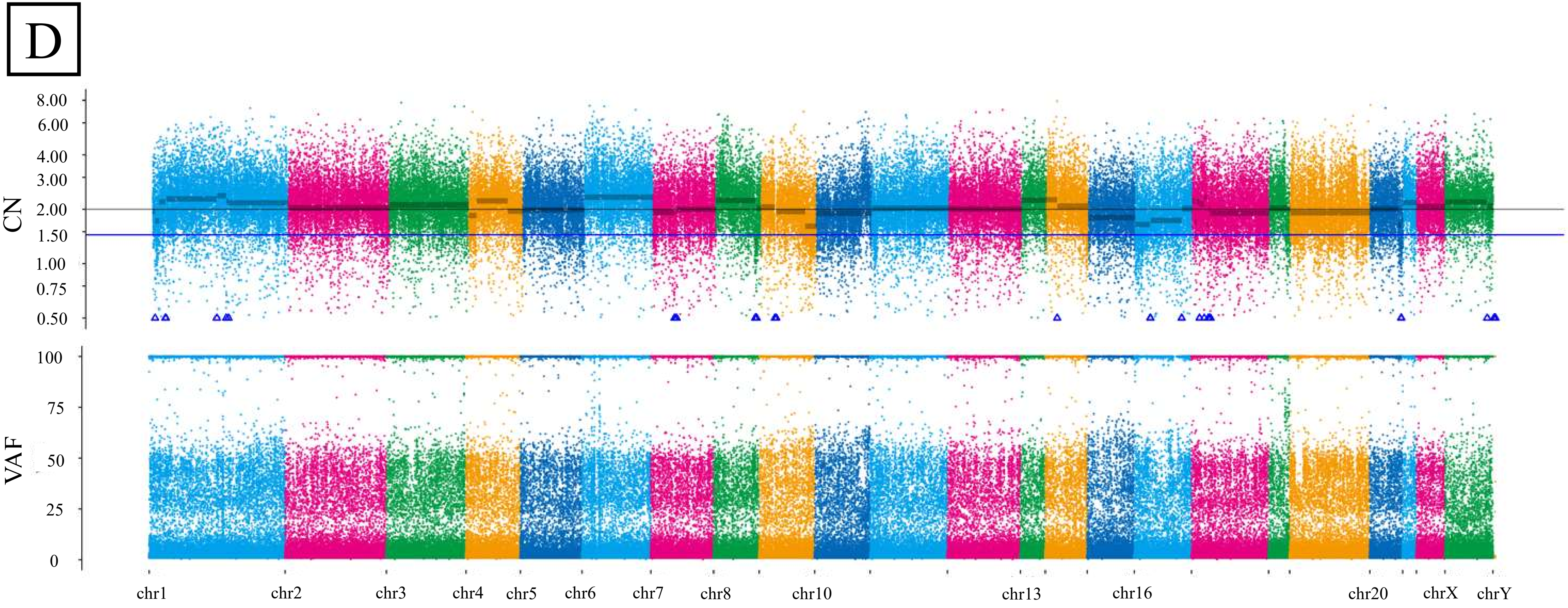

D
CN
8.00
6.00
4.00
3.00
2.00
1.50
1.00
0.75
0.50
VAF
100
75
50
25
0
chr1
chr2
chr3
chr4
chr5
chr6
chr7
chr8
chr10
chr13
chr16
chr20
chrX
chrY

Supplementary Figure Legends

Supplementary Figure S1.

Kaplan–Meier analysis of PFS (A) and OS (B) between the p53 wild-type and p53 mutant groups.

Kaplan–Meier analysis of PFS (C) and OS (D) between the ARID1A (+) and ARID1A (−) groups.

Kaplan–Meier analysis of PFS (E) and OS (F) between the fascin (+) and fascin (−) groups.

Kaplan–Meier analysis of PFS (G) and OS (H) between the E-cadherin (+) and E-cadherin (−) groups.

PFS, progression-free survival; OS, overall survival.

Supplementary Figure S2. Mutation signatures.

(A) Signature 6 exhibits the highest similarity with the mutation profile of the WC in case 2.

(B) Signature 20 exhibits the highest similarity with the mutation profile of the UC in case 2.

(C) Signature 6 exhibits the highest similarity with the mutation profile of the WC in case 3.

(D) Signature 14 exhibits the highest similarity with the mutation profile of the UC in case 3.

WC, well-differentiated component; UC, undifferentiated component.

Supplementary Figure S3. Copy number alteration plot.

The horizontal axis represents chromosome location, and the vertical axis represents gene copy number.

(A) CNA plot of the WC in case 2.

(B) CNA plot of the UC in case 2.

(C) CNA plot of the WC in case 3.

(D) CNA plot of the UC in case 3.

CN, copy number; CNA, copy number alteration; VAF, variant allele frequency;

WC, well-differentiated component; UC, undifferentiated component.
